# Pharmacodynamic and pharmacokinetic behavior of landiolol during dobutamine challenge in healthy adults

**DOI:** 10.1186/s40360-020-00462-x

**Published:** 2020-11-25

**Authors:** Günther Krumpl, Ivan Ulč, Michaela Trebs, Pavla Kadlecová, Juri Hodisch

**Affiliations:** 1 MRN Medical Research Network GmbH, Postgasse 11/22, A-1010 Vienna, Austria; 2 Center for Pharmacology and Analysis (CEPHA) s.r.o, Plzeň, Czech Republic; 3 AOP Orphan Pharmaceuticals AG, Vienna, Austria; 4 Aixial s.r.o., Brno, Czech Republic

**Keywords:** Cardioselective β-blocker, Landiolol, Pharmacokinetics, Pharmacodynamics, Dobutamine

## Abstract

**Background:**

To study the pharmacokinetic and -dynamic behavior of landiolol in the presence of dobutamine in healthy subjects of European ancestry.

**Methods:**

We conducted a single-center, prospective randomized study in 16 healthy subjects each receiving an infusion of dobutamine sufficient to increase heart rate by 30 bpm followed by a 60 min infusion of 10 μg/kg/min landiolol.

**Results:**

Dobutamine-induced increases in heart rate were stable for at least 20 min before a 60 min landiolol- infusion was started. The dobutamine effects were rapidly antagonized by landiolol within 16 min. A further slight decrease in heart rate during 20–60 min of the landiolol infusion occurred as well. Upon termination of landiolol infusion, heart rate and blood pressure recovered rapidly in response to the persisting dobutamine infusion but did not return to the maximum values before landiolol infusion. The pharmacokinetic parameters of landiolol in presence of dobutamine showed a short half-life (3.5 min) and a low distribution volume (0.3 l/kg). No serious adverse events were observed.

**Conclusion:**

Landiolol can antagonize the dobutamine-induced increases in heart rate and blood pressure in a fast way. A rapid bradycardic effect until steady-state plasma levels is followed by a slow heart rate reduction. The latter can be attributed to an early desensitization to dobutamine. Consequently, after termination of landiolol, the heart rate did not achieve maximum pre-landiolol values. The pharmacokinetics of landiolol during dobutamine infusion are similar when compared to short- and long-term data in Caucasian subjects. Landiolol in the given dose can thus serve as an antagonist of dobutamine-induced cardiac effects.

**Trial registration:**

Registration number 2010–023311-34 at the EU Clinical Trials Register, registration date 2010-12-21.

## Background

β-adrenoceptor antagonists are effective regulators of heart rate (HR) and sinus rhythm in postoperative atrial fibrillation and flutter [1–3]. Landiolol is an ultrashort-acting intravenous (i.v.) β_1_-adrenoceptor antagonist with high cardioselectivity (β_1_/β_2_ ratio 255) and low toxicity [4, 5]. Landiolol inhibits the positive chronotropic effects of catecholamines on the heart, where β_1_-receptors are predominantly located. Like other β-blockers, landiolol is thought to reduce the sympathetic drive, resulting in HR reduction, decrease in spontaneous firing of ectopic pacemakers, as well as decelerating the electrical conduction of the atrioventricular node and prolongating its refractory period; thereby exerting antiarrhythmic and anti-ischemic effects [6–9]. In contrast to esmolol, landiolol neither blocks Na, Ca and K channels nor decreases plasma renin levels and thus exerts less effect on left ventricular function and blood pressure [10–13]. In clinical trials, landiolol reduced HR within 1–6 min of administration to patients with tachyarrhythmia during or after surgery, as well as in non-perioperative settings [8, 9, 14–37]. The effect was short-lived after bolus administration, but was sustained during continuous administration of the drug, disappearing quickly (within 5 to 30 min) after completion of administration [5, 8–10, 14–37]. Therefore, landiolol can be considered a highly adjustable and easily titratable β_1_-blocker.

Dobutamine is an intravenous β-adrenoceptor agonist (predominantly β_1_) with an inotropic effect. This sympathomimetic drug is commonly used as a cardiac stimulant in the treatment of heart failure and cardiogenic shock. It is also used in cardiac stress testing to help identify coronary artery disease, as an alternative to physical exercise in patients who cannot perform routine workout in a satisfactory manner [38]. Because of its short half-life (2 min), dobutamine is administered as a continuous intravenous infusion. The co-administration of dobutamine with β-blockers has been described during stress testing as an intervention to speed up termination of the tachycardic effect [39] as well as to enhance the accuracy of the procedure [40]. Concomitant administration has also been described to reduce exaggerated tachycardic effects of dobutamine [41].

Our prospective cross-over study in healthy Caucasian volunteers focused on the investigation of the pharmacodynamic characteristics of a permanent infusion of landiolol or esmolol during dobutamine challenge. The aim of this paper is to describe for the first time the pharmacodynamic and -kinetic behavior of landiolol during dobutamine challenge as a main part of the study; the results obtained with esmolol will be presented separately.

## Methods

### Materials

Landiolol hydrochloride (Onoact® 50 mg lyophilized powder, Ono Pharmaceutical Co., Ltd., Osaka, Japan) was reconstituted to a final concentration of 12 mg/ml with isotonic saline (B. Braun AG, Melsungen, Germany) prior to use. Dobutamine (ADMEDA 250 solution for infusion, 50 ml; concentration: 5 mg/ml, Admeda Arzneimittel GmbH, Nienwohld, Germany) was diluted to 1 mg/ml with sterile isotonic saline prior to use.

### Study population

Sixteen healthy volunteers (as assessed by medical history, physical examination, electrocardiogram (ECG), 2D-echocardiography, hematology, coagulation, clinical chemistry, serology, urinalysis and testing for drug abuse at screening) of European ancestry were recruited for the study. Inclusion criteria were an age of 18–45 years (inclusive), a body-mass index of 18.5–30.0 kg/m^2^ (inclusive), non-smoking, ex-smoking (complete stop of smoking for at least 3 months) or mild smoking (9 cigarettes or less per day). Exclusion criteria were, among others, a history or presence of clinically relevant cardiovascular, renal, hepatic, ophthalmic, pulmonary, neurological, metabolic, hematological, gastrointestinal, endocrine, immunological, psychiatric or skin diseases, hypersensitivity to landiolol, esmolol, dobutamine or related drugs, as well as pregnancy and/or breast-feeding. Individuals with inappropriate vascular anatomy (small, badly visible or invisible veins) were not eligible for the study.

### Study conduct

Our prospective, double-blinded, randomized, two-period, two-treatment crossover study aimed at comparing the short-term pharmacokinetics, pharmacodynamics and tolerability of landiolol with that of esmolol in the continuous presence of the adrenergic stimulant dobutamine in healthy volunteers, was conducted between March and December 2011 at the Center for Pharmacology and Analysis (CEPHA s.r.o.), Plzeň, Czech Republic. Participants were assigned to one of two treatment sequences (landiolol/esmolol or esmolol/landiolol) using a predefined 1:1 randomization scheme. The duration of Periods 1 and 2 was 2 days each, with confinement from at least 11 h before until at least 8 hours after the end of study drug administration and a washout phase of 2 days minimum between periods. The end-of-study examination was performed within 72 h after the end of infusion.

Three indwelling catheters were placed into the cubital veins of each subject in each period. One catheter was used for dobutamine infusion and the other at the same arm for study drug infusion. Blood sampling was performed from the third catheter on the arm contralateral to the study drug administration site. Drug administration and sampling sites were switched between periods. Study subjects were required to be in bed rest and supine position for safety reasons from the start of dobutamine infusion until 2 hours after the end of study drug administration.

Dobutamine infusion was initiated at 10 μg/kg/min for 10 min and incremented by 5 μg/kg/min every 10 min until the targeted HR increase of at least 30 beats per minute (bpm) above baseline or a maximum dose of 30 μg/kg/min were reached. Once the target HR was established, dobutamine administration was continued at an unchanged rate for another 20 min before landiolol (dose: 10 μg/kg/min) was administered for 60 min. Dobutamine infusion was continued at an unchanged rate during and after β-blocker infusion until the HR had returned to a maximum value or to the value before β-blocker infusion or until 60 min after the end of the β-blocker infusion whichever occurred first.

### Pharmacodynamic measurements

Blood pressure and heart rate measurements were undertaken in short intervals to study the specific effects of the drug infusions. Specifically, the ECG was monitored continuously from 10 min before initiation of dobutamine infusion until at least 120 min after the end of β-blocker infusion. ECG parameters (PQ, QRS, QT) were checked throughout at regular intervals. Blood pressure (measured by the cuff method on the arm used for blood sampling) and HR (from the signal of the bedside ECG monitor) were watched closely, and scheduled recordings of systolic and diastolic blood pressure (SBP and DBP, resp.) values were taken at the start of dobutamine infusion, at 2, 4, 6, and 10 min of each dobutamine dose, at 2, 4, 6, 10, 15 and 20 min of the maintenance dobutamine dose, and at the start (0) of and at 4, 8, 16, 28, 44, and 60 min during study drug infusion. Further recordings were taken 4, 8, 16, 28, 44, 60, 75, 90, 105, and 120 min after discontinuation of β-blocker administration. HR values were recorded at the time points for blood sampling and at 15 min intervals thereafter until 120 min after discontinuation of β-blocker infusion. Blood samples were collected at time 0 (before the infusion start), and at 2, 4, 6, 8, 12, 16, 20, 28, 36, 44, 60 (end of infusion), 62, 64, 66, 68, 72, 76, 80, 88, 96, 104, 120 min and at 3, 5, 7, and 9 h after the start of the infusion.

### Assessment of tolerability and safety

Local tolerability was assessed at the start and at the end of β-blocker infusion, and 2 h after the end of infusion as described earlier [42]. For the assessment of safety and tolerability, clinically relevant abnormalities in physical examination, vital signs, ECG, laboratory parameters, as well as local tolerability results and adverse events were assessed or recorded.

### Analytical procedure

Concentrations of landiolol and its metabolites (LM1, LM2) in supernatants of ethanol-precipitated whole blood were quantitated using a validated HPLC-MS/MS method as described earlier [42]. The lower and upper limits of quantitation with this method are 10 ng/ml and 550 ng/ml, resp., for landiolol and LM2, and 50 ng/ml and 2800 ng/ml, resp., for LM1. Intermediate precision (RSD) across the range of quantitation was < 5, < 7 and < 6% for landiolol, LM1 and LM2, resp. Accuracy across the range of quantitation was between 99.6 and 102.1%, 95.7 and 99.3%, and 98.0 and 99.2% for landiolol, LM1 and LM2, resp. Stability of ethanol-precipitated whole blood samples has been shown for 23 h at bench-top temperature, and for 38 weeks when stored at <− 70 °C. Analytes in processed samples are stable for 168 h under auto-sampler conditions (5 °C).

### Pharmacokinetic analysis

Standard pharmacokinetic parameters for landiolol and its metabolites were estimated using non-compartmental methods with the validated software package PhoenixTM WinNonlin® (version 6.1, Pharsight Corp., St. Louis, MO, USA).

### Pharmacodynamic analysis

Time courses of the pharmacodynamic variables were evaluated in terms of absolute values and changes from baseline on an individual basis and by using descriptive statistics for each data point.

### Statistical analysis

Statistical analyses were performed using the software package SAS (version 9.2, SAS Institute Inc., Cary, NC, USA). Segmental analysis with linear least-square regression was performed with the R package ‘segmented’ to compare the two parts of the curve in Fig. 3a. Linear least-square regression analysis in Fig. 3b was performed in MS Excel.

## Results

### Study population

The baseline demographic characteristics of the study population are presented in Table 1.

**Table 1:** Baseline demographics of the study population (*n* = 16)

Demographic variable	
Gender, *n* (%)	
Female	8 (50)
Male	8 (50)
Age, yr (mean ± SD)	33.1 ± 8.2
Weight, kg (mean ± SD)	75.9 ± 8.3
Height, cm (mean ± SD)	176.9 ± 7.0
BMI, kg · m^− 2^ (mean ± SD)	24.2 ± 1.7
Smoking habits, *n* (%)	
Non-smoker	12 (75)
Smoker	4 (25)

*SD* Standard deviation

### Maintenance dobutamine dose

The mean administration rate of dobutamine was 15.3 μg/kg/min.

### Pharmacokinetics

**Fig. 1:**
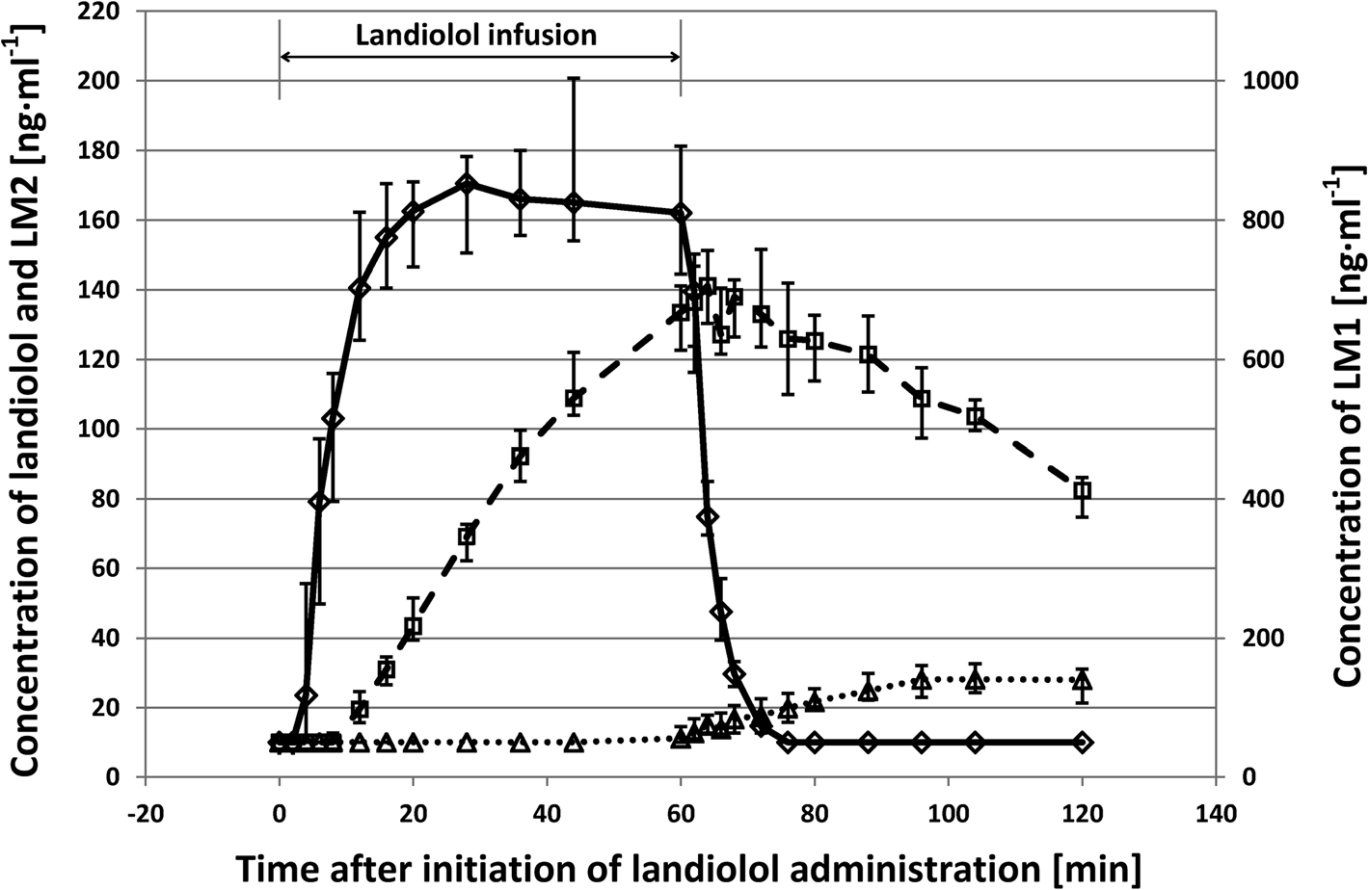
Time courses of the concentrations of landiolol and its metabolites LM1 and LM2 in whole blood when administered for 60 min on top of continuous dobutamine infusion in healthy volunteers (*n* = 16). Median values are shown. Solid line and diamonds: landiolol; broken line and squares: LM1; dotted line and triangles: LM2

The pharmacokinetics (PK) of landiolol and its metabolites LM1 and LM2 in the continuous presence of dobutamine was evaluated during and until 2 h after infusion for 60 min. The time courses of the concentrations of the analytes in venous blood are shown in Fig. 1. Landiolol concentrations increased fast after a lag phase of about 4 min due to the necessary circulation time. Steady state was reached after 4 half-lives, i.e., after 16 min. No significantly different changes in the concentrations of landiolol were observed thereafter until the end of infusion. The concentration of landiolol declined fast after the termination of the infusion. Concentrations of LM1 started to rise with a lag time necessary to overcome the detection limit. LM1 then increased continuously until a few minutes after the end of infusion when convertible landiolol was still present in the blood. Thereafter LM1 followed a slow decay. The first non-zero concentration values of LM2, which is formed from LM1, were recorded at 44 min after the start of infusion. LM1 reached the highest concentration (Cmax) of the three analytes (LM1: 740 ng · ml-1, landiolol: 190 ng · ml-1 and LM2: 30 ng · ml-1; all values medians).

The pharmacokinetic parameters of landiolol and its metabolites were analyzed using compartmental and noncompartmental methods. The results obtained by noncompartmental analysis gave the best fit and are summarized in Table 2. The maximum concentration of landiolol in blood (190 ng · ml^− 1^, geometric mean) was observed at a median t_max_ of 36 min. The terminal elimination half-life of landiolol was 3.5 min. Both metabolites, which are known to be pharmacologically inactive [37], showed a substantially longer half-life than the parent compound (98 and 220 min, resp.). Exposure (based on both C_max_ and AUC_0-∞_) was highest to LM1, followed by the parent drug landiolol and LM2. LM2 concentrations, being formed from LM1, still increased when landiolol had disappeared from the bloodstream.

**Table 2:** PK parameters (non-compartmental analysis) of landiolol and its metabolites during 60 min infusion on top of continuous dobutamine infusion in healthy volunteers of European ancestry (*n* = 16)

Parameter	Statistic	Landiolol	LM1	LM2
Cmax (ng · ml^− 1^)	geo mean (SD)	188 (1.20)	738 (1.16)	30 (1.30)
	[95% CI]	[170, 208]	[681, 799]	[26, 35]
tmax (min)	median	36	64	100
	[range]	[12, 60]	[60, 72]	[90, 180]
AUC0-t (ng · hr. · ml^−1^)	geo mean (SD)	160 (1.18)	1710 (1.34)	82 (1.93)
	[95% CI]	[147, 175]	[1460, 2000]	[58, 117]
AUC0-∞ (ng · hr. · ml^−1^)	geo mean (SD)	162 (1.17)	1920 (1.26)	203 (1.58)
	[95% CI]	[148, 176]	[1700, 2180]	[143, 289]
t1/2 (min)	geo mean (SD)	3.5 (1.15)	98 (1.25)	220 (1.87)
	[95% CI]	[3.2, 3.7]	[87, 110]	[130, 350]
λz (min^−1^)	geo mean (SD)	0.20 (1.15)	0.0071 (1.25)	0.0032 (1.87)
	[95% CI]	[0.19, 0.22]	[0.0063, 0.0080]	[0.0020, 0.0052]
CL (ml · kg^−1^ · min^−1^)	geo mean (SD)	62 (1.17)	5.2 (1.26)	49.2 (1.58)
	[95% CI]	[57, 67]	[4.58, 5.9]	[34.6, 69.8]
VD (ml · kg^−1^)	geo mean (SD)	309 (1.28)	733 (1.15)	15,300 (1.41)
	[95% CI]	[271, 352]	[679, 791]	[11,800, 20,000]

*Cmax* Maximum blood concentration, *tmax* Time until maximum blood concentration, *AUC0-t* Area under the blood concentration-time curve from start of administration to the last measurable concentration estimate, *AUC0-∞* Area under the blood concentration-time curve from start of administration to infinity, *λz* Terminal elimination rate constant, *t1/2* Elimination half-life during the terminal phase, *CL* Total body clearance, *VD* Volume of distribution, *SD* Standard deviation, *95% CI* 95% confidence interval

### Pharmacodynamics

**Fig. 2:**
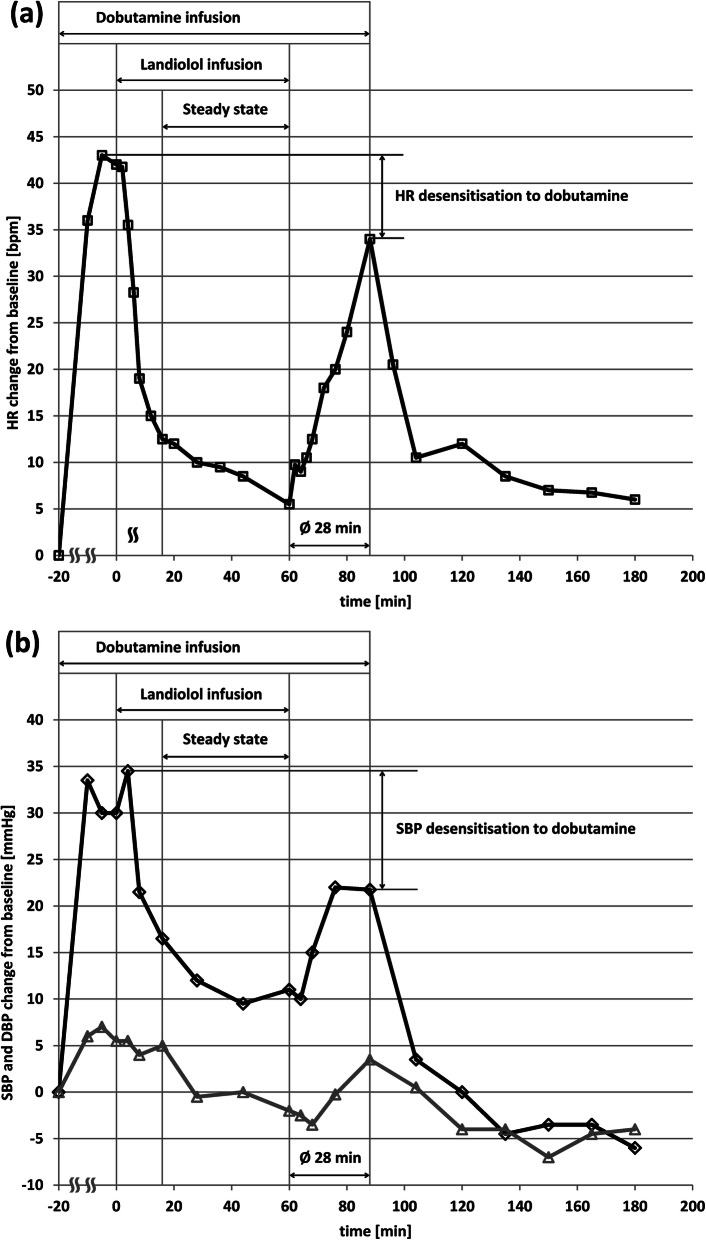
Time courses of changes from baseline for heart rate (**a**) and systolic and diastolic blood pressure (**b**) during and after infusion of landiolol for 60 min on top of continuous dobutamine infusion in healthy volunteers (*n* = 16). Median values are shown

Values of HR and blood pressure (both SBP and DBP) at each time point after initiation of landiolol infusion (on top of continuous dobutamine stimulation) are presented in Table 3 (absolute values) and Fig. 2 (changes from baseline). The dobutamine-induced HR increase was (+ 42 [28, 76] bpm). Shortly after initiation of landiolol infusion, HR started to decrease, and this decrease continued for the whole administration period, with two clearly distinguishable phases. The maximum HR reduction brought about by landiolol at 10 μg/kg/min was − 40 bpm and thus numerically very close to the dobutamine-induced increase. The initial phase of this reduction was quite steep (− 32 bpm within 16 min, which corresponds to 4 times the half-life of landiolol). The second phase was much shallower (− 8 bpm) and lasted for more than 40 min until the end of landiolol infusion.

**Table 3:** Time courses of heart rate, systolic and diastolic blood pressure during and after infusion of landiolol for 60 min on top of continuous dobutamine infusion in healthy volunteers of European ancestry (*n* = 16)

Time (min)	HR [IQR] (bpm)	SBP [IQR] (mmHg)	DBP [IQR] (mmHg)
baseline	65.5 [59.3, 72.5]	107.0 [100.0, 112.3]	72.0 [68.0, 75.5]
-10	101.0 [91.8, 113.0]	137.5 [125.8, 152.8]	76.0 [71.8, 80.5]
-5	107.5 [100.8, 117.1]	133.0 [126.3, 150.1]	77.0 [72.8, 83.0]
0	112.0 [100.5, 120.8]	134.0 [123.0, 145.5]	75.0 [69.8, 85.0]
2	112.5 [102.4, 117.5]	–	–
4	103.5 [97.8, 109.8]	139.5 [124.8, 151.3]	77.0 [68.8, 85.0]
6	92.5 [88.0, 99.5]	–	–
8	85.5 [79.1, 91.0]	126.0 [121.5, 137.3]	77.0 [72.0, 82.5]
12	83 [79.4, 87.3]	–	–
16	79.8 [72.0, 84.3]	122.5 [114.5, 135.0]	74.5 [70.0, 79.8]
20	79.3 [76.5, 82.0]	–	–
28	77.5 [72.3, 78.3]	121.5 [111.0, 129.8]	74.0 [65.5, 79.3]
36	75.5 [71.5, 82.3]	–	–
44	73.0 [70.0, 78.3]	119.0 [110.8, 124.3]	71.0 [65.5, 75.3]
60	71.0 [69.0, 74.3]	121.5 [112.0, 124.3]	73.5 [66.0, 79.8]
62	75.0 [70.9, 81.8]	–	–
64	75.5 [68.8, 79.3]	118.0 [114.8, 124.3]	74.0 [62.8, 79.5]
66	75.8 [69.8, 87.5]	–	–
68	78.5 [70.0, 86.9]	122.5 [114.5, 134.5]	68.5 [63.8, 76.0]
72	85.0 [77.5, 90.0]	–	–
76	91.0 [76.8, 98.5]	128.3 [121.3, 135.5]	70.3 [64.5, 79.3]
80	89.5 [82.8, 104.6]	–	–
88	105.0 [94.8, 111.6]	122.8 [117.3, 143.0]	72.0 [69.6, 79.8]
96	86.5 [75.5, 99.5]	–	–
104	78.0 [71.0, 84.0]	109.5 [101.8, 116.5]	73.0 [63.5, 77.3]
120	80.8 [70.0, 82.0]	106.5 [99.8, 114.3]	69.5 [62.8, 74.3]
135	74.5 [65.0, 81.8]	104.5 [96.3, 109.3]	66.5 [61.5, 74.0]
150	71.5 [65.5, 81.0]	106.0 [95.0, 112.0]	67.0 [62.5, 75.0]
165	75.0 [66.3, 82.9]	105.0 [97.5, 109.3]	66.5 [62.8, 74.3]
180	72.5 [65.5, 82.8]	102.0 [98.0, 112.3]	67.5 [62.5, 73.0]

*IQR* Interquartile range

**Fig. 3:**
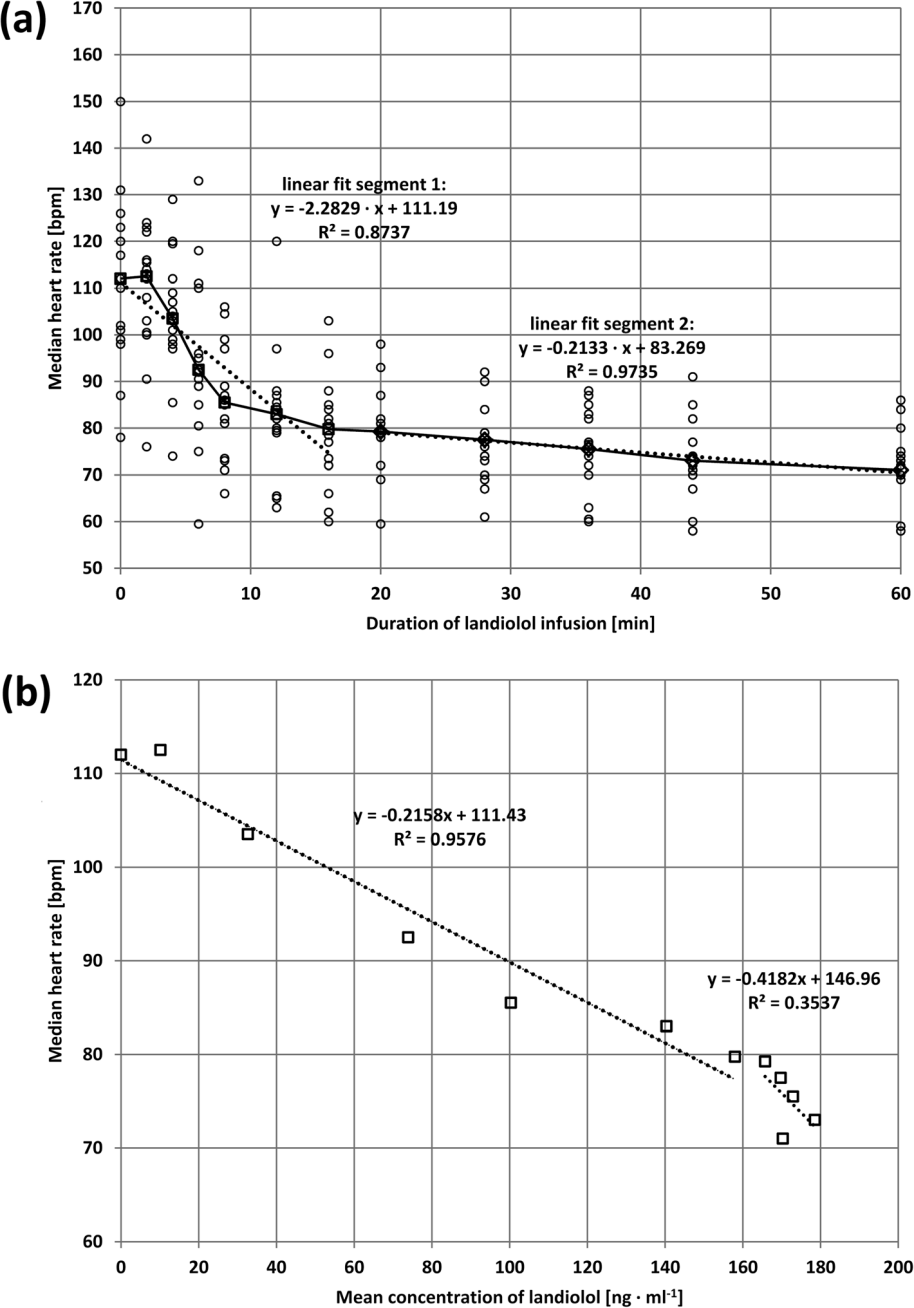
Regression analyses of heart rate versus duration of landiolol infusion (**a**) and heart rate versus concentrations of landiolol in venous blood (**b**)

Figure 3 shows the HR effects using a segmental regression analysis versus time (a) and landiolol concentration in blood (b). The HR time course suggests a distinct separation of two phases which were significantly different when compared statistically using segmental analysis (Fig. 3a). The relationship between blood concentrations of landiolol and HR reduction (Fig. 3b) is different. Regression analysis shows a reasonable linear fit between 20 and 160 ng/ml, i.e., when steady state plasma concentration was achieved at 80 bpm. Thereafter, below 80 bpm (that is, between 20 and 60 min), the linear fit between blood concentrations of landiolol and HR gets lost with R2 being 0.35 (Fig. 3b) and a slope approaching infinity which indicates the contribution of effects with markedly different time constants.

The recovery from the bradycardic effect after termination of landiolol infusion occurred very fast, and the maximum was achieved after 28 min (Fig. 2a). This is in line with the rapid decrease of landiolol concentrations in blood due to its half-life of 3.5 min (Fig. 1, Table 2). It is evident from Fig. 2a that HR recovery was incomplete since the post-infusion maximum value was 105 bpm, as opposed to 112.5 bpm at the initiation of landiolol administration. After dobutamine discontinuation, a fast recovery to values below 80 bpm occurred within 15 min; but HR values were still above baseline (72.5 bpm vs 65 bpm) even at the end of the observation period.

The changes in SBP values from the start of dobutamine infusion until two hours after the end of landiolol infusion showed a similar pattern to the changes in HR. DBP was only slightly elevated by dobutamine and slightly decreased by landiolol (Fig. 2b). It is interesting to note that blood pressure values returned to levels below baseline shortly after discontinuation of dobutamine administration, as opposed to HR that remained above baseline until the end of the observation period.

### Safety and tolerability

A total of 26 adverse events (AEs) occurred in 8 subjects; 9 (35%) of these being cardiac disorders (palpitations, ventricular extrasystoles and ventricular tachycardia), 9 (35%) being nervous system disorders (headache, paresthesia), 3 (15%) being gastrointestinal disorders (nausea, vomiting) and 3 (15%) being general disorders (pressure sensation, feeling hot). 62% of these reactions occurred during the initial dobutamine infusion, and 38% during dobutamine and concomitant landiolol infusion. Two cases of moderate headache of which one was already present in the dobutamine phase were considered related to landiolol. Two non-zero local tolerability scores (mild tenderness at the beginning and at the end of landiolol infusion, resp.) were observed in 1 out of 16 subjects.

## Discussion

### Pharmacodynamics

After initiation of landiolol infusion, the dobutamine-induced HR decreased continuously over the infusion period of one hour, with a substantial part of the pharmacodynamic effect (− 32 bpm) observed already in the early phase (until 16 min) even if landiolol was not administered as a bolus. A rapid onset of the HR-reducing effect had been also seen in the absence of dobutamine in a comparative study versus esmolol [42]. A further slow decrease of HR (− 8 bpm) was observed after 16 min despite the fact that concentrations of landiolol in blood did not change during this steady-state phase. Regression analysis addressing the correlation between HR and administration time clearly differentiated two bradycardic processes. This was supported by regression analysis between HR and concentrations of landiolol in blood (Fig. 3b). Interestingly a small dobutamine-induced heart rate elevation (5 bpm) remained during landiolol infusion. This phenomenon was seen before (41) and can be explained by a β2-mediated stimulation of the sinus node which cannot be antagonized by a highly selective β1-receptor antagonist.

Previous investigations with long-term landiolol infusions had shown stable HR and blood concentrations between 16 and 60 min and thereafter [42]. Similarly, it has been reported previously that dobutamine concentrations in blood are also quite stable during a 60 min infusion [43]. One might speculate that landiolol may have reduced the concentrations of dobutamine in blood in the later phase. This is however unlikely since both agents have comparable half-lives (2–4 min) and are metabolized differently as landiolol is cleaved by blood and tissue esterases [44], whereas dobutamine is inactivated in tissues and liver [45]. On the contrary, one would rather expect that a reduction of liver perfusion due to landiolol-induced β_1_-antagonism would reduce the elimination of dobutamine and consequently increase its effect through elevation of the blood concentrations. A dobutamine-induced elevation of landiolol blood concentrations through competing elimination mechanisms would be an alternative explanation. However, different elimination pathways, independency of liver status in the elimination of landiolol [46, 47] and, as a direct evidence, constant landiolol concentrations in blood from minute 16 onwards speak against such an interaction.

We assume that the later phase of HR reduction is thus not induced by landiolol but rather reflecting the desensitization of the dobutamine effects in the background. This assumption is supported by the findings in the recovery phase. Shortly after the end of landiolol infusion, HR started to increase again but did not reach pre-landiolol values despite continued dobutamine infusion. The maximum HR (achieved after 28 min) was more than 10 bpm lower than the maximum pre-landiolol value. This, in line with the slow HR reduction during the second landiolol infusion phase, can thus be explained as consequence of a dobutamine-induced downregulation of the β_1_-receptor function, a desensitization phenomenon already described experimentally and clinically in previous investigations [48–51].

The changes in SBP and - to a lesser extent - DBP over the observation period were similar to the effect on HR (Table 3, Fig. 2b). Interestingly, SBP and DBP were below the baseline values after dobutamine discontinuation which supports the explanation above and further suggests a possible α-receptor downregulation (DBP) during dobutamine infusion. The persistently increased HR after dobutamine infusion can thus be explained by a reflex increase of the sympathetic drive to counteract the persistent blood pressure decrease due to desensitization.

Comparison of the hemodynamic results in the present study with those obtained in an earlier study [42] reveals an interesting differential effect. In the study conducted under resting conditions, landiolol-induced SBP reduction was less pronounced than the effect on HR. In the present study the effects on SBP and HR were similar, indicating that landiolol is able to completely antagonize the effect of a β_1_-adrenergic stimulus on the cardiovascular system, a characteristic that may turn out beneficial for the early termination of cardiovascular effects after dobutamine stress testing.

Antagonism of dobutamine-induced tachycardia has been described for esmolol, another short-acting β-blocker using bolus application [39, 52]. The dose of 10 μg/kg/min landiolol used in the present study produced blood concentrations which correspond to the ones achieved with landiolol bolus doses of 0.1 mg · kg^− 1^ [53]. As this dose induced an onset of the bradycardic effect within 1 min, it can be assumed that bolus application can reduce the time to maximum HR reduction from the 16 min observed in the current study to few minutes when a rapid termination of the tachycardic dobutamine effect after stress testing is warranted.

### Pharmacokinetics

The pharmacokinetic results on landiolol and its inactive metabolites were similar to what has been described before in Asian and Caucasian subjects [5, 16, 37, 42, 53]. Specifically, the lack of a substantial change in the pharmacokinetic parameters of landiolol during dobutamine infusion confirms that changes in liver perfusion do not affect (and specifically do not speed up) the elimination of landiolol. The absence of an impact of the liver-status and -perfusion on the half-life of landiolol was described previously [46, 47] as was the absence of gender effects ([37], unpublished data of [54]).

### Safety and tolerability

As non-selective and β-blockers with low β_1_-selectivity may cause coronary spasms after dobutamine stress echocardiography [52], a β-blocker with increased selectivity like landiolol (β_1_/β_2_ ratio = 255) may have a lower likelihood to expose dobutamine α-receptor effects through blockade of vasodilating β_2_-receptors. In line with this, no serious adverse events were observed in our study. 16/26 adverse events occurred before administration of landiolol and were likely related to dobutamine. Landiolol showed very good local and systemic tolerability and an excellent safety profile, although it has to be kept in mind that this was a small group of healthy individuals. Nevertheless, this result is essentially in line with the safety profile reported from earlier studies in Asian or Caucasian subjects [5–9, 15, 19, 23, 37, 42, 53, 54].

## Conclusion

When dobutamine at a mean dose of 15 μg/kg/min is used to increase HR and SBP, the short-acting β-blocker landiolol at 10 μg/kg/min can almost completely antagonize these effects within 16 min. Thereafter, these variables further decrease slowly over 40 min, which, in the presence of a constant landiolol concentration in the blood, likely reflects an early desensitization to dobutamine effects. The reduced agonistic effect of dobutamine is also observed after discontinuation of landiolol infusion, as both the maximum HR and SBP effects were not reached anymore. Dobutamine does not alter the pharmacokinetic parameters of landiolol and its metabolites. Landiolol infusion during dobutamine challenge is tolerated well with a low incidence of adverse events.

## Data Availability

The datasets used and analysed during the current study are available from the corresponding author on reasonable request.

## Abbreviations

AE: Adverse event
AUC: Area under the curve
bpm: Beats per minute
CI: Confidence interval
CL: Total body clearance
DBP: Diastolic blood pressure
ECG: Electrocardiogram
HR: Heart rate
IQR: Interquartile range
RSD: Relative standard deviation
SBP: Systolic blood pressure
SD: Standard deviation
VD: Volume of distribution

## References

[1] Andrews TC, Reimold SC, Berlin JA, Antman EM (1991). Prevention of supraventricular arrhythmias after coronary artery bypass surgery. A meta-analysis of randomized control trials. Circulation.

[2] Kowey PR, Taylor JE, Rials SJ, Marinchak RA (1992). Meta-analysis of the effectiveness of prophylactic drug therapy in preventing supraventricular arrhythmia early after coronary artery bypass grafting. Am J Cardiol.

[3] Crystal E, Connolly SJ, Sleik K, Ginger TJ, Yusuf S (2002). Interventions on prevention of postoperative atrial fibrillation in patients undergoing heart surgery: a meta-analysis. Circulation.

[4] Iguchi S, Iwamura H, Nishizaki M, Hayashi A, Senokuchi K, Kobayashi K (1992). Development of a highly cardioselective ultra short-acting β-blocker, ONO-1101. Chem Pharm Bull(Tokyo).

[5] Atarashi H, Kuruma A, Ino T, Saitoh H, Onodera T, Ono T (1993). ONO-1101: a new ultra-short acting β-adrenergic blocker: initial study of efficacy, safety and pharmacokinetics. Cardiovasc Drugs Ther.

[6] Taenaka N, Kikawa S (2013). The effectiveness and safety of landiolol hydrochloride, an ultra-short-acting β1-blocker, in postoperative patients with supraventricular tachyarrhythmias: a multicenter, randomized, double-blind, placebo-controlled study. Am J Cardiovasc Drugs.

[7] Jinzaki M, Hirano M, Hara K, Suzuki T, Yamashina A, Ikari Y (2013). A randomized, double-blind, placebo-controlled, phase II dose-finding study of the short acting β_1_-blocker, landiolol hydrochloride, in patients with suspected ischemic cardiac disease. Int J Cardiovasc Imaging.

[8] Yoshiya I, Ogawa R, Okumura F, Shimada Y, Hanaoka K (1997). Clinical evaluation of landiolol hydrochloride (ONO-1101) on perioperative supraventricular tachyarrhythmia - a phase III, double-blind study in comparison with placebo. Rinsho Iyaku (Jpn J Clin Ther Med).

[9] Yoshiya I, Ogawa R, Okumura F, Shimada Y, Hanaoka K (2000). Clinical evaluation of an ultra short acting β_1_-blocker: landiolol hydrochloride (ONO-1101), on perioperative supraventricular tachyarrhythmia - a double-blind, dose finding study (late phase II study). Rinsho Iyaku (Jpn J Clin Ther Med).

[10] Sasao J, Tarver SD, Kindscher JD, Taneyama C, Benson KT, Goto H (2001). In rabbits, landiolol, a new ultra-short-acting β-blocker, exerts a more potent negative chronotropic effect and less effect on blood pressure than esmolol. Can J Anaesth.

[11] Iizuka T, Kakinuma T, Hamada Y, Isshiki A (2004). A novel ultra short acting beta-blocker, landiolol supress the central sympathetic nerve activity directory and exerts a more potent negative chronotropic effect and less effect on blood pressure than esmolol. Eur J Anesthesiol.

[12] Sugiyama A, Takahara A, Hashimoto K (1999). Electrophysiologic, cardiohemodynamic and beta-blocking actions of a new ultra-short-acting beta-blocker, ONO-1101, assessed by the in vivo canine model in comparison with esmolol. J Cardiovasc Pharmacol.

[13] Ikeshita K, Nishikawa K, Toriyama S, Yamashita T, Tani Y, Yamada T (2008). Landiolol has a less potent negative inotropic effect than esmolol in isolated rabbit hearts. J Anesth.

[14] Yamazaki A, Kinoshita H, Shimogai M, Fujii K, Nakahata K, Hironaka Y (2005). Landiolol attenuates tachycardia in response to endotracheal intubation without affecting blood pressure. Can J Anaesth.

[15] Xiao J, He P, Zou Q, Zhao Y, Xue Z, Deng X (2015). Landiolol in the treatment of the intraoperative supraventricular tachycardia: a multicenter, randomized, double-blind, placebo-controlled study. J Clin Anesth.

[16] Murakami M, Furuie H, Matsuguma K, Wanibuchi A, Kikawa S, Irie S (2005). Pharmacokinetics and pharmacodynamics of landiolol hydrochloride, an ultra short-acting β_1_-selective blocker, in a dose escalation regimen in healthy male volunteers. Drug Metab Pharmacokinet.

[17] Saito S, Nishihara F, Akihiro T, Nishikawa K, Obata H, Goto F (2005). Landiolol and esmolol prevent tachycardia without altering cerebral blood flow. Can J Anaesth.

[18] Sugiura S, Seki S, Hidaka K, Masuoka M, Tsuchida H (2007). The hemodynamic effects of landiolol, an ultra-short-acting β_1_-selective blocker, on endotracheal intubation in patients with and without hypertension. Anesth Analg.

[19] Hanada K, Higuma T, Nishizaki F, Sukekawa T, Yokota T, Yamada M (2012). Randomized study on the efficacy and safety of landiolol, an ultra-short-acting β1-adrenergic blocker, in patients with acute myocardial infarction undergoing primary percutaneous coronary intervention. Circ J.

[20] Hasuo H, Tomiyasu S, Hojo M, Fujigaki T, Fukusaki M, Sumikawa K (1998). Effect of ONO-1101, a novel short-acting β-blocker on hemodynamic responses to isoflurane inhalation and tracheal intubation. J Anesth.

[21] Higuchi H (2010). Feasibility of continuous intravenous administration of landiolol for acute myocardial infarction: first clinical experience and its safe directions for use. J Am Coll Cardiol.

[22] Ito S, Konishi T, Imazuru T, Matsuzaki K, Jikuya T (2011). Comparative study between landiolol and amiodarone for therapeutic efficacy after open heart surgery. Kyobu Geka (Jpn J Thorac Surg).

[23] Ito N, Tashiro T, Morishige N, Nishimi M, Hayashida Y, Minematsu N (2012). Safety and efficacy of an ultrashort-acting β1-blocker on left ventricular dysfunction. Heart Surg Forum.

[24] Kawaguchi M, Kawaraguchi Y, Yamamoto Y, Hayashi H, Abe R, Inoue S (2010). Effects of landiolol on systemic and cerebral hemodynamics and recovery from anesthesia in patients undergoing craniotomy. J Anesth.

[25] Kitamura A, Sakamoto A, Inoue T, Ogawa R (1997). Efficacy of an ultrashort-acting β-adrenoceptor blocker (ONO-1101) in attenuating cardiovascular responses to endotracheal intubation. Eur J Clin Pharmacol.

[26] Morishima A, Hirao S, Nagasaka S, Yokoyama S, Kaneda K, Nishiwaki N (2009). Suppressive effect of landiolol hydrochloride on atrial fibrillation following surgical repair of acute type a aortic dissection. Jpn J Vasc Surg.

[27] Nagai R, Kinugawa K, Inoue H, Atarashi H, Seino Y, Yamashita T (2013). Urgent management of rapid heart rate in patients with atrial fibrillation/flutter and left ventricular dysfunction: comparison of the ultra-short-acting β1-selective blocker landiolol with digoxin (J-land study). Circ J.

[28] Nagaoka E, Arai H, Tamura K, Makita S, Miyagi N (2014). Prevention of atrial fibrillation with ultra-low dose landiolol after off-pump coronary artery bypass grafting. Ann Thorac Cardiovasc Surg.

[29] Nakanishi K, Takeda S, Kim C, Kohda S, Sakamoto A (2013). Postoperative atrial fibrillation in patients undergoing coronary artery bypass grafting or cardiac valve surgery: intraoperative use of landiolol. J Cardiothorac Surg.

[30] Nojiri T, Yamamoto K, Maeda H, Takeuchi Y, Funakoshi Y, Maekura R (2011). Efficacy of low-dose landiolol, an ultrashort-acting β-blocker, on postoperative atrial fibrillation in patients undergoing pulmonary resection for lung cancer. Gen Thorac Cardiovasc Surg.

[31] Sakamoto A, Kitakaze M, Takamoto S, Namiki A, Kasanuki H, Hosoda S (2012). Landiolol, an ultra-short-acting β_1_-blocker, more effectively terminates atrial fibrillation than diltiazem after open heart surgery: prospective, multicenter, randomized, open-label study (JL-KNIGHT study). Circ J.

[32] Sezai A, Minami K, Nakai T, Hata M, Yoshitake I, Wakui S (2011). Landiolol hydrochloride for prevention of atrial fibrillation after coronary artery bypass grafting: new evidence from the PASCAL trial. J Thorac Cardiovasc Surg.

[33] Sezai A, Nakai T, Hata M, Yoshitake I, Shiono M, Kunimoto S (2012). Feasibility of landiolol and bisoprolol for prevention of atrial fibrillation after coronary artery bypass grafting: a pilot study. J Thorac Cardiovasc Surg.

[34] Taenaka N, Kikawa S (2013). Dose-dependent effect of landiolol, a new ultra-short-acting β_1_-blocker, on supraventricular tachyarrhythmias in postoperative patients. Clin Drug Investig.

[35] Uehara K, Ueyama K, Ito H, Fukuda T, Sasaki K, Abe T (2010). Clinical analysis of precaution against atrial fibrillation following cardiac surgery: Landiolol or amiodarone?. Kyobu Geka (Jpn J Thorac Surg).

[36] Sakamoto A, Hamasaki T, Kitakaze M (2014). Perioperative landiolol administration reduces atrial fibrillation after cardiac surgery: a meta-analysis of randomized controlled trials. Adv Ther.

[37] Plosker GL (2013). Landiolol: a review of its use in intraoperative and postoperative tachyarrhythmias. Drugs.

[38] Krahwinkel W, Ketteler T, Gödke J, Wolfertz J, Ulbricht LJ, Krakau I (1997). Dobutamine stress echocardiography. Eur Heart J.

[39] Abdullah EE, Pollick C (1997). Symptomatic and hemodynamic recovery following dobutamine stress echo: benefit of low-dose esmolol administration. Int J Card Imaging.

[40] Weissman NJ, Levangie MW, Guerrero JL, Weyman AE, Picard MH (1996). Effect of beta blockade on dobutamine stress echocardiography. Am Heart J.

[41] Kijima Y, Yoshima S, Takagi T, Asai M, Lee K, Bando K (2017). Concomitant administration of landiolol and dobutamine in acute heart failure syndrome with atrial tachyarrhythmia. J Card Fail.

[42] Krumpl G, Ulč I, Trebs M, Kadlecová P, Hodisch J, Maurer G (2018). Pharmacokinetics and pharmacodynamics of low-, intermediate-, and high-dose landiolol and esmolol during long-term infusion in healthy whites. J Cardiovasc Pharmacol.

[43] Ahonen J, Aranko K, Livanainen A, Maunuksela E-L, Paloheimo M, Olkkola KT (2008). Pharmacokinetic-pharmacodynamic relationship of dobutamine and heart rate, stroke volume and cardiac output in healthy volunteers. Clin Drug Investig.

[44] Suno M, Kunisawa T, Yamagishi A, Ono T, Yamamoto J, Yamada T (2009). Detection of landiolol using high-performance liquid chromatography/fluorescence: a blood esterase-sensitive ultra-short-acting β_1_ receptor antagonist. J Chromatogr B Analyt Technol Biomed Life Sci.

[45] SmPC Dobutamine. https://www.medicines.org.uk/emc/product/6462/smpc. Accessed: 2020 May 06.

[46] Takahata T, Yasui-Furukori N, Sakamoto J, Suto K, Suto T, Tateishi T (2005). Influence of hepatic impairment on the pharmacokinetics and pharmacodynamics of landiolol hydrochloride, an ultra-short-acting beta1-blocker. Drugs R D.

[47] Matsumoto N, Aomori T, Kanamoto M, Usui T, Shiga T, Yamamoto K (2012). Influence of hemodynamic variations on the pharmacokinetics of landiolol in patients undergoing cardiovascular surgery. Biol Pharm Bull.

[48] Puy Portillo M, del Barrio AS, García-Calonge MA, Martínez JA (1996). Desensitization effect of in vivo treatment with metaproterenol on β1, β2 and β3-responsiveness in rat adipocytes. Life Sci.

[49] Marion-Latard F, de Glisezinski I, Crampes F, Berlan M, Galitzky J, Suljkovicova H (2001). A single bout of exercise induces β-adrenergic desensitization in human adipose tissue. Am J Physiol Regul Integr Comp Physiol.

[50] Welsh RC, Warburton DER, Humen DP, Taylor DA, McGavock J, Haykowsky MJ (2005). Prolonged strenuous exercise alters the cardiovascular response to dobutamine stimulation in male athletes. J Physiol.

[51] Unverferth DA, Blanford M, Kates RE, Leier CV (1980). Tolerance to dobutamine after a 72-hour continuous infusion. Am J Med.

[52] Mateo Martínez A, Martínez Pasqual del Riquelme M, López Ayala JM, Saura Espín D (2015). Coronary vasospasm after dobutamine stress echocardiogram triggered by esmolol. Int J Cardiol.

[53] Krumpl G, Ulc I, Trebs M, Kadlecová P, Hodisch J (2016). Pharmacokinetics and pharmacodynamics of two different landiolol formulations in a healthy Caucasian group. Eur J Pharm Sci.

[54] Stix G, Wolzt M, Domanovits H, Kadlecová P, Husch B, Trebs M (2020). Open-label two-dose pilot study of landiolol for the treatment of atrial fibrillation/atrial flutter in Caucasian patients. Circ J.

